# Insights into organelle forming RNAs: Diversity, functions and future perspectives

**DOI:** 10.1002/ame2.70080

**Published:** 2025-11-28

**Authors:** Meng Gong, Xiangting Wang, Xiaolin Liang

**Affiliations:** ^1^ Division of Life Sciences and Medicine, Department of Geriatrics, Gerontology Institute of Anhui Province, The First Affiliated Hospital University of Science and Technology of China Hefei Anhui China; ^2^ The RNA Institute University of Science and Technology of China Hefei Anhui China; ^3^ Division of Biomedical Sciences, MOE Key Laboratory for Membraneless Organelles and Cellular Dynamics, Hefei National Science Center for Physical Sciences at Microscale, School of Life Sciences University of Science and Technology of China Hefei Anhui China; ^4^ Centre for Leading Medicine and Advanced Technologies of IHM University of Science and Technology of China Hefei Anhui China

**Keywords:** non‐coding RNAs, organelle formation RNAs, *TubAR*, *LoNA*, *NEAT1*, *MALAT1*

## Abstract

RNA molecules play diverse and essential roles in cellular processes beyond their well‐known functions in gene expression and regulation. While ribosomal RNAs (rRNAs) have long been recognized as structural components of ribosomes, recent research has highlighted the importance of a distinct group of RNAs which directly compose the structures or organelles in mammalian cells. We refer to these as ‘organelle formation RNAs’. Specifically, the discovery of tubulin‐associated lncRNA (*TubAR*), the first identified cytoskeleton‐forming RNA, has expanded our understanding of RNA functionality; we now recognize ‘organelle formation RNAs’ not only as regulatory molecules but also as direct structural components within cellular subunits. Other ‘organelle formation RNAs’ include paraspeckle‐forming RNAs, nuclear speckle‐forming RNAs, and nucleolus‐forming RNAs. Various RNAs contribute to the formation of distinct cellular structures, while also participating in intricate intermolecular interactions. Understanding these molecules not only uncovers their fundamental roles in cellular physiology but also suggests potential applications in the treatment of related diseases. By examining the latest advancements and methodologies in organelle formation RNA research, this review provides a comprehensive overview of the intricate mechanisms of these RNAs and future directions in the field.

## Graphical abstract

RNA molecules play crucial roles in the formation and maintenance of cellular structures and organelles. These ‘organelle formation RNAs’ include ribosomal RNAs, paraspeckle‐forming RNAs, nuclear speckle‐forming RNAs, nucleolus‐forming RNAs, and cytoskeleton‐forming RNA. This review explores their structural characteristics, functions, and potential implications for health and disease.

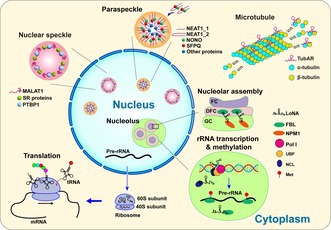

## 1 INTRODUCTION

In the central dogma of molecular biology, three types of macromolecules—DNA, RNA, and proteins—form the core pathway for the flow of genetic information, ensuring the normal functioning and transmission of genetic material in living organisms.^1^, ^2^ RNA not only serves as a bridge in this process but also has emerged as a focal point of research due to its diversity and intricate functionalities throughout evolution. RNA performs a wide array of essential functions, from carrying genetic information and catalyzing biochemical reactions to regulating gene expression, all of which are crucial for cellular processes.

**FIGURE 1:**
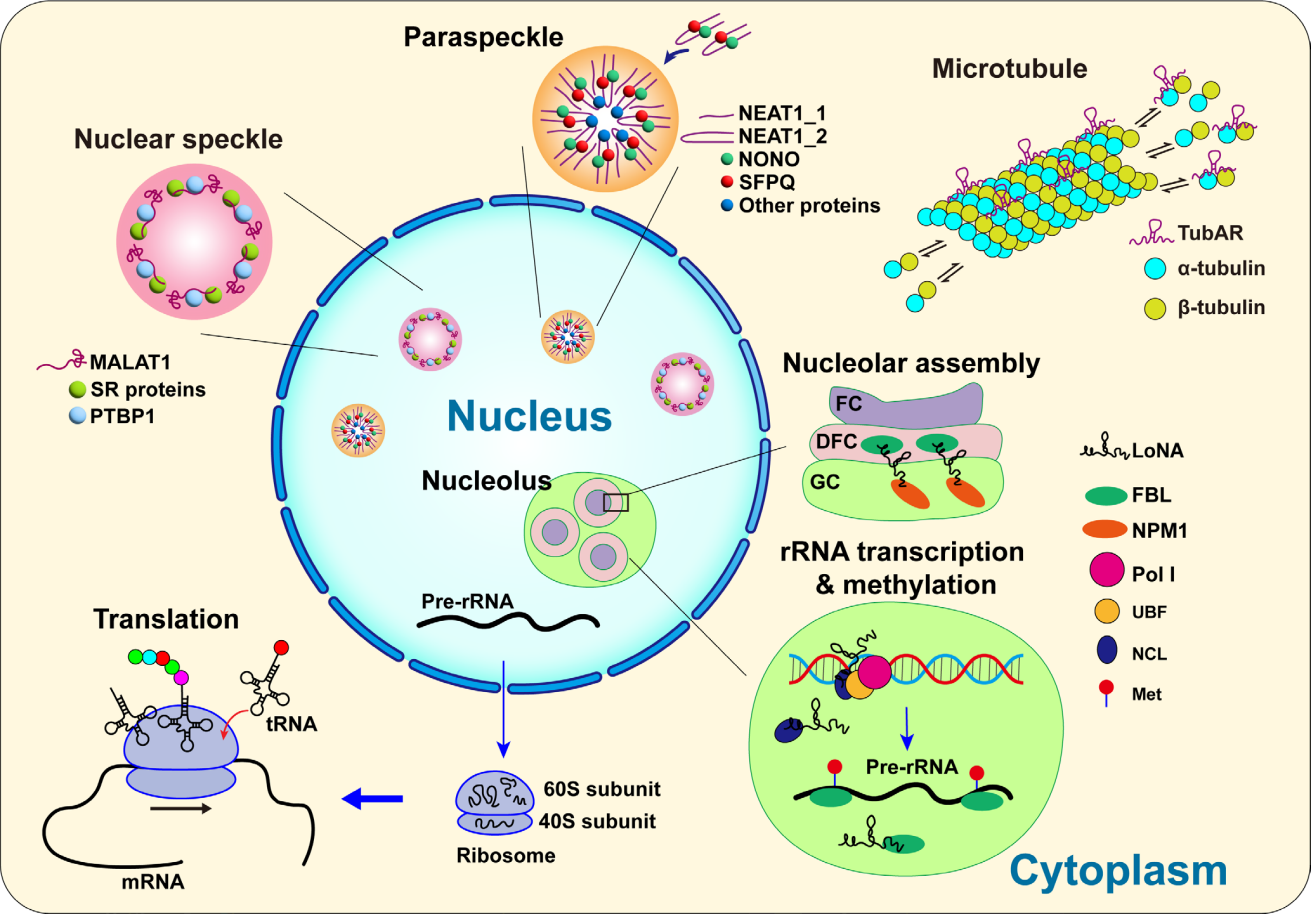
Classification and functions of organelle formation RNAs. (1) *NEAT1_2* acts as the scaffold for paraspeckle assembly by binding NONO and SFPQ to form a core RNP complex, driving phase separation, while *NEAT1_1* localizes to paraspeckles but is dispensable for their formation. (2) Eukaryotic rRNA (*18S* in *40S*; *28S/5.8S/5S* in *60S*) provides ribosome structure and catalytic function for protein synthesis. (3) *TubAR*, a microtubule‐associated lncRNA, directly binds tubulin isotypes TUBA1A and TUBB4A to facilitate microtubule assembly. (4) *MALAT1* interacts with SR proteins, PTBP1 and other proteins in nuclear speckles to regulate alternative splicing and RNA processing. (5) *LoNA* inhibits rRNA transcription by binding NCL to disrupt UBF/Pol I recruitment to rDNA, while reducing rRNA methylation through FBL competition; it also mediates nucleolar assembly by facilitating FBL‐NPM1 interactions to establish FC/DFC/GC compartmentalization via phase separation.

Beyond these functions, a distinct subset of RNAs plays an essential role in the architectural assembly and structural integrity of specific subcellular compartments. We define ‘organelle formation RNAs’ as RNAs whose primary biological function involves serving as essential structural scaffolds or organizers for the formation, maintenance, or spatial organization of cellular structures, while acknowledging that some (e.g., *MALAT1*, *LoNA*, etc.) additionally perform regulatory roles. This definition serves to distinguish them from RNAs whose predominant function is regulatory (e.g., gene silencing, transcriptional activation). These RNAs encompass a spectrum of molecules including rRNAs,^3^, ^4^, ^5^, ^6^ paraspeckle forming RNAs,^7^, ^8^, ^9^ nuclear speckle formation RNA,^10^, ^11^ nucleolus forming RNA,^12^, ^13^ and cytoskeleton formation RNA^14^ (Figure 1). Each of these RNA types contributes uniquely to cellular processes such as translational regulation, stress response, cell differentiation, RNA processing and splicing, rRNA maturation, and cytoskeleton assembly.

Long non‐coding RNAs (lncRNAs) are well known for their crucial regulatory functions in gene expression, both at the transcriptional and post‐transcriptional stages.^15^, ^16^, ^17^ However, a subset of lncRNAs also functions as structural molecules, influencing cellular activities through their three‐dimensional architecture. Notably, lncRNAs like nuclear enriched abundant transcript 1 (*NEAT1*),^18^ metastasis associated lung adenocarcinoma transcript 1 (*MALAT1*),^18^ nucleolus‐specific lncRNA (*LoNA*),^13^ and *TubAR* ^14^ exemplify this structural role. These lncRNAs underscore the diverse mechanisms by which lncRNAs can impact cellular functions beyond gene regulation, highlighting their importance as structural components in cellular biology.

## 2 rRNA: CATALYSTS AND STRUCTURAL COMPONENTS OF RIBOSOME

rRNAs are essential components of ribosomes, the cellular organelles responsible for protein synthesis.^3^, ^19^ In eukaryotes, ribosomal genes encode distinct rRNAs for the large subunit (*28S*, *5.8S*, and *5S* rRNAs) and the small subunit (*18S* rRNA).^20^ The *18S*, *5.8S*, and *28S* rRNAs originate from a common *45S* pre‐rRNA transcript, while the *5S* rRNA is encoded independently.^3^, ^21^, ^22^ Within the ribosome, rRNA serves both structural and functional roles.^23^ Specifically, rRNA molecules act as catalysts, facilitating the chemical reactions involved in protein synthesis. The large subunit rRNA, especially the *28S* rRNA in eukaryotes (*23S* rRNA in prokaryotes), carries out the peptidyl transferase activity critical for catalyzing peptide bond formation.^3^, ^24^

Modifications within rRNA are crucial for its function; for instance, alterations such as N^6^‐dimethyladenosine (m^6^Am)^6^ or N^6^‐methyladenosine (m^6^A) modifications in *18S* rRNA cause decreased 40S subunit availability and reduced protein synthesis.^25^, ^26^ Similarly, the m^5^C modification in *28S* rRNA, introduced by NSUN5, regulates ribosome activity, and its disruption leads to impaired ribosomal function and reduced protein synthesis.^27^, ^28^, ^29^ The *5.8S* rRNA plays a crucial role in ribosome translocation, thereby contributing to the process of protein synthesis.^30^, ^31^ Additionally, the key role of *5S* rRNA is assembly of the large ribosomal subunit.^32^

During translation initiation, the small subunit rRNA plays an essential role in identifying the correct start codon on mRNA (usually AUG) and positioning it properly within the ribosome.^33^, ^34^, ^35^ This process ensures that the ribosome starts synthesizing the protein from the correct site. The interaction between the small subunit rRNA and the initiator tRNA (modified methionine) helps establish the translational reading frame.^33^ Additionally, throughout the elongation phase of translation, both ribosome subunit rRNAs contribute to the accurate pairing of codons on mRNA with complementary anticodons on tRNA.^3^, ^36^, ^37^ This process guarantees the accurate addition of amino acids to the growing nascent polypeptide chain in alignment with the mRNA sequence.^35^

In summary, rRNA's dual role as both a structural scaffold and the catalytic engine of the ribosome, with its function regulated by specific modifications, is essential to maintaining ribosome structure and function.

## 3 *NEAT1* : STRUCTURAL FOUNDATION OF PARASPECKLE

*NEAT1*, first identified in 1997 and characterized in 2007, is transcribed from a MEN1‐associated locus on chromosome 11.^9^, ^38^ *NEAT1* is a 4 kb‐long, minimally spliced RNA that exhibits widespread expression in both humans and mice.^9^ *NEAT1* is predominantly localized in the nucleus, where it appears to avoid both the nucleoli and heterochromatin during the interphase of cell division. Interestingly, *NEAT1* forms several prominent, bright foci, which are often dispersed throughout the nucleus, and these have now been characterized as a classical liquid–liquid phase separation phenomenon.^9^

In 2009, it was discovered that *NEAT1* produces two ncRNA isoforms.^7^ *NEAT1_1*, which is 3.7 kb in length and contains a 3′ poly(A) tail, overlaps with the 5′ end sequence of *NEAT1_2* (22.7 kb). *NEAT1_2* lacks a 3′ poly(A) tail, and the 3′ end triple helix generated after processing by RNase P maintains its structural stability.^39^, ^40^, ^41^ Inhibiting the recognition and usage of *NEAT1* proximal polyadenylation sites (PASs) can promote the expression of *NEAT1_2* and reduce the formation of *NEAT1_1*.^41^, ^42^

*NEAT1* knockdown disrupts the formation of membrane‐less organelles and reduces their assembly. In particular, *NEAT1_2* is crucial for this process, with its middle domain playing a key role in paraspeckle assembly.^7^, ^8^ It's the primary structural scaffold of paraspeckles.^40^ The essential paraspeckle proteins NONO and SFPQ bind to *NEAT1_2*, forming a *NEAT1_2*‐RNP that serves as an intermediate in paraspeckle formation (Figure 1). This initiates oligomerization with other paraspeckle proteins, such as FUS and TDP43, and promotes the formation of the phase‐separated paraspeckle structure.^8^, ^43^, ^44^ Although *NEAT1_1* is present in paraspeckles, it is not essential for paraspeckle formation.^39^, ^41^

These findings establish *NEAT1* as a critical component in paraspeckle formation, making it the first RNA identified as ‘organelle formation RNA’ in scientific history.

Under cellular stress, such as viral infections or DNA damage, increased *NEAT1* expression enhances paraspeckle formation and functionality to support physiological demands and stress responses.^8^, ^45^, ^46^, ^47^ Dysregulation of *NEAT1* and paraspeckles formation have been linked to a range of diseases, such as cancer and neurodegenerative disorders, highlighting their pivotal roles in pathological states and offering potential avenues for therapeutic intervention.^39^, ^45^, ^48^, ^49^, ^50^, ^51^ Targeting *NEAT1's* alternative polyadenylation (APA) processing can inhibit platinum resistance in ovarian cancer. Blocking CSTF3‐mediated generation of the short isoform *NEAT1_1* can attenuate platinum resistance in OC cells.^42^

## 4 *MALAT1* : ESSENTIAL COMPONENT OF NUCLEAR SPECKLES AND THE HiNoCo BODY

*MALAT1*, also known as *NEAT2*, is a lncRNA overexpressed in metastatic non‐small‐cell lung cancer, with higher levels linked to increased metastasis and potential involvement in cancer progression.^52^, ^53^, ^54^ Like *NEAT1_2*, *MALAT1* lacks a 3′ poly(A) tail and uses a stable RNA triplex structure at the 3′ end to protect against rapid exonucleolytic degradation.^55^

*MALAT1* is predominantly localized in the nucleus, where it is concentrated in dynamic subnuclear structures known as nuclear speckles, which play a key role in RNA processing and splicing.^9^ *MALAT1* plays a crucial role in modulating these processes by interacting with a range of proteins, including splicing factors like SR proteins, PTBP1, and other RNA‐binding proteins.^56^, ^57^ These interactions are crucial for modulating alternative splicing patterns and influencing gene expression, thus impacting cellular function and homeostasis.^10^, ^58^, ^59^ One of the primary functions of *MALAT1* is acting as a scaffold to facilitate the recruitment of YTHDC1 to nuclear speckles.^58^ This interaction, especially the ability of YTHDC1 to recognize *MALAT1*'s m6A modifications, is indispensable for this process.^58^ However, recent studies found that knockout of *MALAT1* has no detectable influence on the formation of nuclear speckles, indicating that other factors also contribute to the integrity of nuclear speckles.^60^ *MALAT1* is not essential for the formation of nuclear speckles, and in response to heat shock *MALAT1* relocates to a distinct nuclear body, the HiNoCo body, which forms near nuclear speckles and is independent of heat shock factor 1 (HSF1).^61^

*MALAT1*'s localization within nuclear speckles is not only critical for RNA processing but also for maintaining the structural integrity of the nucleus.^58^, ^59^ Dysregulation of *MALAT1* expression or its interactions with nuclear speckles has been linked to aberrant splicing events and altered gene expression profiles, which are commonly observed in diseases, including cancer.^10^, ^11^ *MALAT1* was upregulated in lenalidomide‐resistant multiple myeloma (MM) cell lines, and the knock down of *MALAT1* significantly reduced MM cell proliferation and viability in both lenalidomide‐sensitive and ‐resistant cells.^62^ Therefore, understanding how *MALAT1* operates within nuclear speckles is essential for deciphering its functional roles in both normal cellular processes and disease pathogenesis.

## 5 *LoNA* : NUCLEOLUS STRUCTURE MAINTAINER

The nucleolus is a dynamic and functionally specialized region within the nucleus, playing a key role in ribosome assembly and the cellular response to stress.^63^, ^64^ It consists of three main regions: the fibrillar center (FC), the dense fibrillar component (DFC), and the granular component (GC).^63^, ^64^ A recent investigation has revealed the existence of an additional region, known as the periphery of the dense fibrillar component (PDFC), located between the DFC and GC.^65^ The biogenesis of ribosomes is a complex, multistep process primarily occurring in the nucleolus.^66^

*LoNA*, a nucleolus‐specific lncRNA that is highly expressed in neurons, can simultaneously regulate both the transcription of rRNA and its post‐transcriptional methylation modifications^13^ (Figure 1). First, it suppresses rRNA transcription by binding to nucleolin (NCL) through its 5′ binding sites, sequestering NCL and thereby impairing the recruitment of UBF and RNA polymerase I (Pol I) to rDNA chromatin. This interaction modifies the epigenetic status of rDNA and ultimately reduces rRNA production. Second, *LoNA* inhibits rRNA methylation by competing with U3 snoRNA for binding to fibrillarin (FBL) via its 3′ box C/D sequence. This competition diminishes FBL's methyltransferase activity, leading to decreased 2’‐O‐methylation at functionally critical rRNA sites. Further research revealed that in the presence of *LoNA*, the methylation frequency at rRNA sites within key functional domains was significantly reduced, which is consistent with the diminished activity of FBL.^13^, ^67^

In addition, *LoNA* also mediates the nucleolus assembly process and influences embryonic development. After meiotic division and fertilization, nucleolus precursor bodies (NPBs) appear in zygotes (1‐cell stage embryos). NPBs gradually change their morphology as the embryo develops, and embryos at the more advanced stages of development contain typical differentiated nucleoli.^68^ The NPB maturation mediated by *LoNA* is essential for embryogenesis beyond the 2‐cell stage. It facilitates the attachment of NPM1, which is enriched in the GC, to FBL, which is concentrated in the DFC, promoting the formation of a compartmentalized nucleolus by supporting the liquid–liquid phase separation of these two nucleolar proteins. Depletion of *LoNA* results in a failure of nucleolar assembly, causing NPM1 to become mislocalized and acetylated in the nucleoplasm. Modified NPM1 can bind to DNA and recruit the PRC2 complex, which catalyzes H3K27me3 modification, thereby repressing gene transcription. This process ultimately leads to embryonic development arrest at the 2‐cell stage.^12^

The expression level of *LoNA* is dependent on neuronal activity, with high expression in the resting state. *LoNA* levels significantly decrease in neurons stimulated with KCL, and behavioral stimuli such as learning and memory tasks can notably reduce *LoNA* expression levels. Specific knockdown of *LoNA* in the hippocampus significantly increases the levels of synaptic‐related proteins, enhances synaptic plasticity, and improves higher cognitive functions.^13^

*LoNA* plays crucial dual roles in both nucleolar organization and neuronal protein synthesis, making it a key player in learning, memory, and neurodegenerative diseases. *LoNA* is a nucleolus‐specific molecule that regulates nucleolar structure through epigenetic modifications and is an important stabilizing factor for nucleolar integrity. Moreover, *LoNA*'s involvement in nucleolar maturation during development highlights its broader importance in embryonic development and cellular differentiation. The multifunctional nature of *LoNA* underscores its significance in both neurobiology and developmental biology.

## 6 *TubAR*: LONG SEARCHING MICROTUBULE REGULATOR AND COMPONENT RNA

Microtubules are fundamental to cellular structure, essential for morphology, transport, d ivision, and viability.^69^ Since 1977, numerous studies have shown that RNase treatment severely disrupts microtubule‐related structures, indicating critical structural roles for specific RNAs.^70^, ^71^, ^72^, ^73^

**FIGURE 2:**
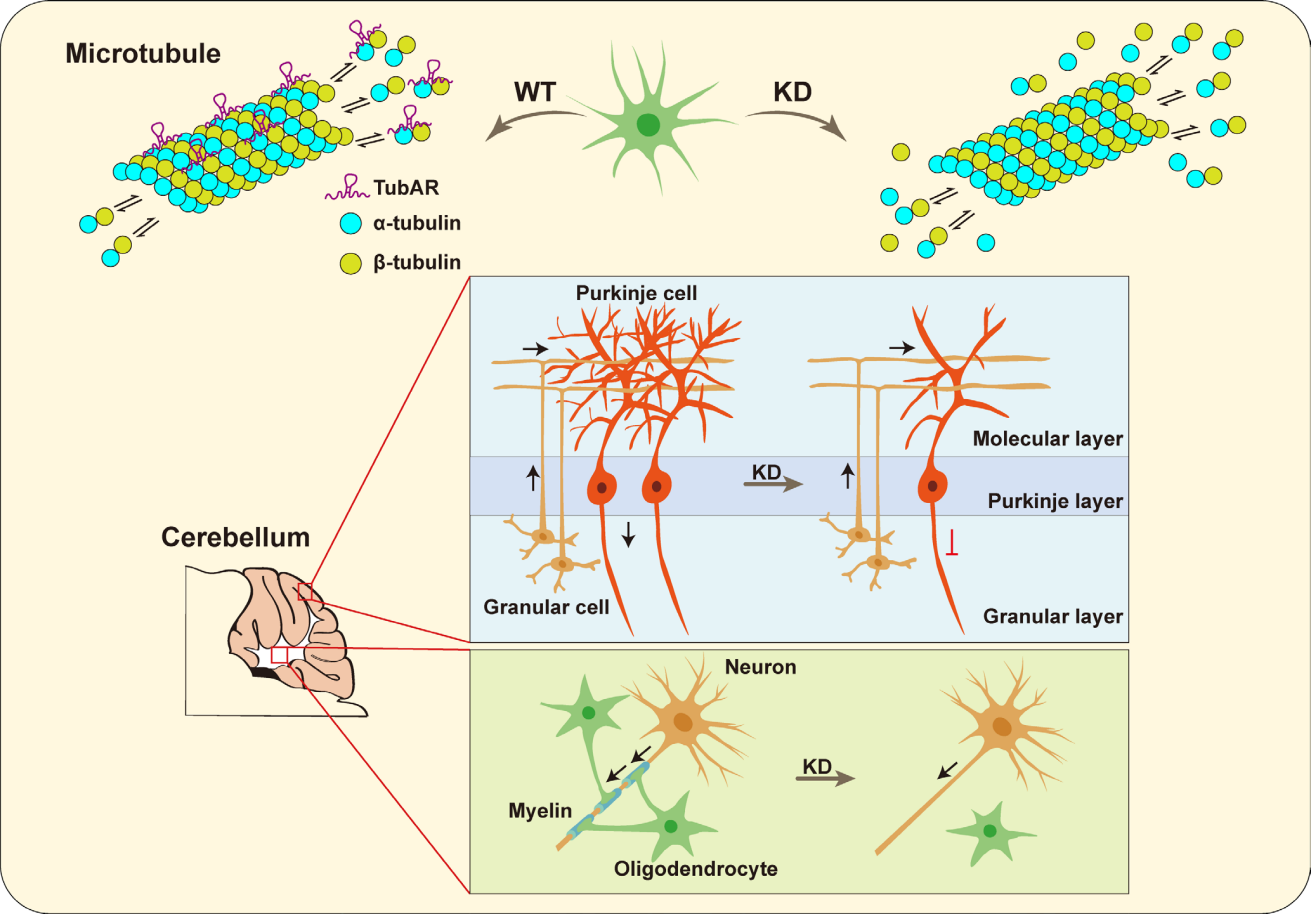
Regulating mechanism of microtubule component RNA *TubAR. TubAR* directly binds to tubulin isotypes TUBA1A and TUBB4A to promote microtubule assembly, and its knockdown disrupts microtubule formation and causes cell death. In the mouse cerebellum, *TubAR* knockdown leads to Purkinje cell loss and reduced myelination.

*TubAR*, a recently identified lncRNA prominently expressed in the cerebellum, stands out for its pivotal role in the organization of the cytoskeleton.^14^ It directly interacts with tubulin isotypes TUBB4A and TUBA1A, forming a complex essential for microtubule assembly.^14^ Researchers have elucidated *TubAR*'s profound influence on microtubule dynamics and cellular integrity. Depletion of *TubAR* disrupts microtubule formation, leading to cellular death, particularly evident in neurons and oligodendrocytes^14^ (Figure 2). Microtubule co‐sedimentation assays demonstrated that in vitro transcribed *TubAR* predominantly co‐pelleted with microtubules during ultracentrifugation, but remained in solution without adding tubulins, proving its direct microtubule‐binding capability. These findings highlight *TubAR*'s unique identity as a ‘microtubule structural RNA’, underscoring its regulatory function in organizing cytoskeletal elements and influencing broader neurological processes.

Interestingly, the above‐cited study found that certain TUBB4A mutations associated with hypomyelination (such as D249N, V255I, R282P, and N414K) fail to interact with *TubAR* and TUBA1A, which correlates with their pathological effects on microtubule dynamics and brain function. In contrast, the non‐hypomyelination‐causing TUBB4A mutation (R2G), which exhibits normal tubulin polymerization and oligodendrocyte morphology, showed constitutive binding to TUBA1A. This suggests that the specific interaction between *TubAR* and TUBB4A‐TUBA1A heterodimers is critical for maintaining microtubule integrity, and disruptions in this interaction may contribute to the pathological mechanisms in certain tubulinopathies. In vivo, *TubAR* knockdown in the mouse cerebellum causes severe demyelination, loss of neurons and glia, and impaired motor function, confirming its vital role in cerebellar development and microtubule‐dependent brain processes.

The discovery of *TubAR* as a key regulatory lncRNA that facilitates microtubule assembly and tubulin heterodimer formation marks a major step forward in unraveling the molecular mechanisms that regulate the cytoskeleton. While lncRNAs are well‐known gene regulators, emerging evidence reveals their additional role as structural components. *TubAR*'s microtubule‐organizing function suggests RNAs may serve as direct architectural elements in the cytoskeleton. This paradigm shift implies RNA could structurally influence other cytoskeletal networks (actin, intermediate filaments) and cellular organization, potentially impacting diverse pathological processes from neurodegeneration to cancer.

## 7 ORGANELLE‐FORMING RNAs IN ANIMAL MODELS: MECHANISTIC AND THERAPEUTIC INSIGHTS

LncRNAs play crucial roles in organizing nuclear architecture and facilitating the formation of membrane‐less organelles through liquid–liquid phase separation. Among these, *NEAT1* and *MALAT1* are well‐characterized architectural RNAs (arcRNAs) that scaffold nuclear paraspeckles and nuclear speckles, respectively. Recent studies in animal models provide profound insights into their functions in health and disease. This review synthesizes key findings on these RNAs in vertebrate models, primarily mice, rats and zebrafish. These models provide indispensable platforms for validating RNA‐centric therapies, including antisense oligonucleotides (ASOs), CRISPR‐based interventions, and RNA editing technologies.

*NEAT1*'s role in paraspeckle assembly directly impacts disease pathogenesis and treatment. In Alzheimer's disease (AD) mouse models (e.g., hAPP‐J20), hippocampal *NEAT1* upregulation correlates with astrocyte reactivity and mitophagy suppression via NEDD4L‐mediated PINK1 degradation. Depleting *NEAT1* rescues cognitive deficits and reduces gliosis, highlighting its potential as a therapeutic target.^74^, ^75^ In MPTP‐induced Parkinson's disease (PD) mice, *NEAT1* promotes autophagy and apoptosis by targeting miR‐107‐5p,^76^ and is involved in PD pathophysiology through the miR‐376b‐3p/NLRP3 signaling pathway.^77^ The upregulation of *NEAT1* expression levels in the MPTP‐induced PD model suggests that ASOs targeting *NEAT1* could offer potential neuroprotection. Beyond neurodegeneration, *NEAT1* deficiency in Kras^−/‐G12D^‐expressing mice enhances oncogene‐driven transformation and promotes the development of premalignant pancreatic intraepithelial neoplasias and cystic lesions, demonstrating its tumor‐suppressive role.^78^

*MALAT1*'s regulation of nuclear speckles and splicing positions it at the nexus of developmental and degenerative diseases. Zebrafish studies reveal that *MALAT1* knockdown causes embryonic lethality and malformations, while radiation‐induced *MALAT1* suppression disrupts splicing fidelity across generations.^79^ In mammalian models, *MALAT1* is upregulated in the retinas of STZ‐induced diabetic rats and db/db mice, and *MALAT1* knockdown alleviates retinal vascular damage in diabetic animals.^80^ In mouse models of breast cancer and other tumors, *MALAT1* promotes metastasis and immune evasion by regulating SERPINB6B, and targeting *MALAT1* with ASOs effectively suppresses tumor metastasis.^81^ In hematological malignancies, the promotion of tumorigenesis and drug resistance by *MALAT1* makes it a potential therapeutic target. In a multiple myeloma (MM) mouse model, targeting *MALAT1* with ASOs significantly reduced tumor burden in the disseminated MM mouse model and notably prolonged the mice's lifespan.^82^

*LoNA* and *TubAR* are emerging players in organelle dynamics, with limited research in animal models. In the APP/PS1 transgenic Alzheimer's disease mouse model, *LoNA* knockdown improves learning and memory.^13^ Meanwhile, *TubAR* deficiency using shRNAs leads to Purkinje cell loss, demyelination, and locomotor deficits in mice.^14^ Future work on *LoNA* and *TubAR* will accelerate the development of precision RNA medicines for neurodegeneration, cancer, and beyond.

## 8 CONCLUSION AND PERSPECTIVE

In conclusion, RNA molecules involved in organelle formation (organelle forming RNAs) are fundamental to cellular function and regulation, playing essential roles in a diverse range of biological processes. ‘RNA world’ hypothesis posits that early life forms relied predominantly on RNA for various regulatory functions. While nowadays most contemporary RNA primarily serves in gene transcription, many still exhibit significant enzymatic activity and structural roles within cells. For instance, some RNA molecules contribute to the microtubule assembly and participate in the organization of cellular cytoskeleton.^14^

Beyond individual functions, the interactions between different RNAs are crucial to their functional network within the cell. *LoNA* inhibits *rRNA* transcription and 2’‐O‐methylation by binding to nucleolin and fibrillarin, reducing their activity and modifying the epigenetic status of rDNA.^13^, ^67^ *MALAT1* and *NEAT1* are neuroprotective molecules induced by hypoxic preconditioning (HPC) and may exert a synergistic effect in neuroprotection, as they reduce NR2B expression, with their knockdown leading to increased NR2B levels.^83^ In breast cancer, the upregulation of *NEAT1* and *MALAT1* is associated with reduced APOBEC3B (A3B) levels and increased APOBEC3A (A3A) activity. RNA binding sequesters A3B, inhibiting its base‐editing activity, and its release from *NEAT1/MALAT1* enhances A3B activity while impairing A3A function.^84^

While significant progress has been made, the study of organelles forming RNA remains an emerging field with many unknowns. Advanced technologies, such as cryo‐electron microscopy, hold great potential for uncovering new insights into the structural and functional roles of RNA in cellular systems.^85^, ^86^ Future developments in these areas will likely provide more comprehensive understanding and open new avenues for exploring how different organelles contribute to RNA function and its involvement in various cellular processes.

Moreover, a large portion of the non‐coding RNA, often dubbed the ‘dark matter’ of the genome, remains poorly understood, yet it is increasingly recognized for its importance in gene expression regulation and cellular dynamics. As technological advances continue to deepen our insight into RNA structure and function, future research will explore the diverse roles and regulatory networks of structural RNAs more deeply. Investigations into the biology of structural RNA are likely to uncover novel therapeutic targets and diagnostic biomarkers across a range of diseases. The integration and application of cutting‐edge techniques, such as high‐throughput sequencing and advanced imaging, will propel our understanding of structural RNA dynamics in both health and pathology. By leveraging these advancements, the field is poised to uncover additional layers of RNA‐mediated regulation and pave the way for transformative discoveries in molecular biology and medicine.

## AUTHOR CONTRIBUTIONS

**Meng Gong:** Investigation; visualization; writing – original draft; writing – review and editing. **Xiangting Wang:** Conceptualization; funding acquisition; supervision; writing – review and editing. **Xiaolin Liang:** Conceptualization; funding acquisition; investigation; writing – original draft; writing – review and editing.

## FUNDING INFORMATION

This work was supported by National Natural Science Foundation of China (32400436 to XLL and 32170557 to XW); Anhui Provincial Key Research and Development Project (2022e07020020 to XW); Research Funds of Centre for Leading Medicine and Advanced Technologies of IHM (2023IHM01034 to XW); and Anhui Postdoctoral Scientific Research Program Foundation (2024C879 to XLL).

## CONFLICT OF INTEREST STATEMENT

The authors declare that they have no known competing interest.

## ETHIC STATEMENT

None.

## Data Availability

Not applicable.
